# Identifying transcription factor and microRNA mediated synergetic regulatory networks in lung cancer

**DOI:** 10.1186/1471-2105-14-S17-A14

**Published:** 2013-10-22

**Authors:** Ramkrishna Mitra, Jingchun Sun, Min Zhao, Zhongming Zhao

**Affiliations:** 1 Department of Biomedical Informatics, Vanderbilt University, Nashville, TN 37232, USA; 2 Department of Psychiatry, Vanderbilt University, Nashville, TN 37232, USA; 3 Department of Cancer Biology, Vanderbilt University, Nashville, TN 37232, USA; 4 Center for Quantitative Sciences, Vanderbilt University School of Medicine, Nashville, TN 37232, USA

## Background

It has been demonstrated that, at the network level, the transcriptional regulation by transcription factors (TFs) and post-transcriptional regulation by microRNAs (miRNAs) are tightly coupled. Aberrant expression of these bio-molecules is linked to several diseases, including lung cancer. In this study, we pursued a regulatory network-based approach mediated by TFs and miRNAs for a comprehensive investigation of gene regulation patterns in lung cancer.

## Materials and methods

We introduced a directional network that corresponds to significantly differentially expressed (DE) miRNAs and genes between lung tumor and matched normal samples. We predicted miRNA targets in genes by parsing TargetScan prediction results. To find the regulation of TF to genes or miRNAs, we explored the TFs and their binding profiles from the TRANSFAC Professional database. TFs are either significantly DE or have target molecules which are significantly enriched in DE subspace. In this approach, a signed edge of the network illustrates potential repression or activation/repression mediated by a miRNA or TF, respectively, to their target molecules.

## Results and conclusions

We obtained a significantly enriched set of TF- miRNA mediated three-node based feed forward loops (FFLs) with signed edges. The edges of the signed three-node FFLs were validated using completely independent data set. The observation of critical miRNAs in the Wnt signaling pathway, with partial verification from previous studies, demonstrates that our network-based approach is promising for the identification of new and important miRNAs and their regulation in lung cancer.

